# Do all inhibitions act alike? A study of go/no-go and stop-signal paradigms

**DOI:** 10.1371/journal.pone.0186774

**Published:** 2017-10-24

**Authors:** Ran Littman, Ádám Takács

**Affiliations:** 1 Department of Clinical Psychology and Addictology, Institute of Psychology, Eötvös Loránd University, Budapest, Izabella utca 46., Hungary; 2 Department of Cognitive Psychology, Institute of Psychology, Eötvös Loránd University, Budapest, Izabella utca 46., Hungary; Southwest University, CHINA

## Abstract

Response inhibition is frequently measured by the Go/no-go and Stop-signal tasks. These two are often used indiscriminately under the assumption that both measure similar inhibitory control abilities. However, accumulating evidence show differences in both tasks' modulations, raising the question of whether they tap into equivalent cognitive mechanisms. In the current study, a comparison of the performance in both tasks took place under the influence of negative stimuli, following the assumption that ''controlled inhibition'', as measured by Stop-signal, but not ''automatic inhibition'', as measured by Go/no-go, will be affected. 54 young adults performed a task in which negative pictures, neutral pictures or no-pictures preceded go trials, no-go trials, and stop-trials. While the exposure to negative pictures impaired performance on go trials and improved the inhibitory capacity in Stop-signal task, the inhibitory performance in Go/no-go task was generally unaffected. The results support the conceptualization of different mechanisms operated by both tasks, thus emphasizing the necessity to thoroughly fathom both inhibitory processes and identify their corresponding cognitive measures. Implications regarding the usage of cognitive tasks for strengthening inhibitory capacity among individuals struggling with inhibitory impairments are discussed.

## Introduction

In various circumstances, alternative courses of action and thoughts have to be inhibited in order to allow the emergence of an adaptive, flexible and goal-directed behavior [1]. Within the fields of neuroscience and cognitive science, inhibitory control is often referred to as a multi-domain executive function that is critical for flexible responsivity to changing task demands and is thereby an essential component of adaptive behavioral regulation [2].

Although the requirement to suppress a dominant response may be present in multiple task contexts such as Stroop interference and Wisconsin Card Sort Testing [3, 4], it is most clearly measured by Go/no–go (GNG) and Stop-signal (SST) paradigms [5]. Both tasks are based on repeated execution of motor response (''go'' response, e.g. key pressing or lever pulling) to visual stimuli, while on some trials a pre-defined visual or auditory ''stop'' signal (or ''no-go'' sign) instructs participants to inhibit their habitual go response [6]. The core difference between the two tasks is often the temporal location of the inhibitory signal within the main motor task [6, 7]. While on a typical GNG task the no-go sign is presented simultaneously with or instead of the go stimulus [8], on SST the stop-signal is presented after the go stimulus, so that the response is already in the process of completion [9].

Despite those differences, a majority of studies define a unitary action-inhibition deficit by using both tasks interchangeably and without providing the methodological rationale behind choosing one over the other [6]. Consequently, it has been argued that the term ''inhibition'' has been overextended and is often broad and inconsistent across authors [4], stressing that researchers need to be more specific when discussing and measuring inhibition-related functions. Furthermore, inhibitory control was suggested to be a heterogeneous construct which consists of multiple kinds of inhibitory processes well as a range of tasks used to measure it [10].

Such conceptualizations are supported by the findings regarding different neuroanatomical and neurochemical processes involved in the operation of each task. While both tasks were found to activate a communal network of brain regions including the anterior cingulate cortex (ACC), the inferior frontal cortex (IFC) and the pre-supplementary motor area (pre-SMA), the pattern of activation tended to be bilateral for GNG, but predominated by right hemisphere in SST [3, 6, 11] (For further discussion regarding the differences between the neural correlations in both tasks see [12]). Additional evidence demonstrated inhibitory impairment in SST performance following damage to the right pre-SMA, while impaired GNG performance was followed by damage to the left, but not the right pre-SMA [12, 13]. In other studies which investigated the connections between inhibition and the activation of neurotransmitters, 5-HT was shown to play a significant role in the inhibitory processes taking place in GNG tasks, but not in SST, while noradrenaline was shown to be influential especially in SST but not in GNG tasks (for a review see [6], also [14]). Such evidence, among others, has raised the question of whether both tasks actually tap into the same cognitive mechanisms, or perhaps into fundamentally different ones [6, 15, 16]. Consequently, several researchers have conceptually differentiated between ''action restraint'' (or automatic, bottom-up inhibition), where the stimulus and the required response are consistently paired and which does not require further executive control (as in GNG), and ''action cancellation'' (or controlled, top-down inhibition), where the stimulus and the required response are inconsistently paired and which relies upon additional executive control (as in SST) [7, 16, 17].

### Negative stimuli and their impact on response and response inhibition performance

When studying the modes of operation of executive functions, emotionally aversive stimuli are often used as an effective tool for impacting performance in various cognitive tasks. Indeed, negative stimuli were shown to interfere with various cognitive functions [18–20], to impair the execution of several response types [21–23] and to increase the levels of noradrenaline and cortisol [24, 25].

Following these findings, several studies have investigated the possible modulation of inhibitory control by emotional stimuli [26, 27]. When studied within the context of inhibitory functions, emotionally negative material was typically reported to have no impact upon the inhibitory measure of commission errors in GNG tasks [28–32], although some counter findings were also reported [33]. However, the impact of negative stimuli over inhibitory functions in SST appears to be less clear, with some studies [34] demonstrating an impairing effect of negative stimuli over inhibitory functions in SST, and others [35, 36] reporting of improved inhibitory performance in SST after the exposure to negative stimuli. Furthermore, it was argued that aversive stimuli of diverse strength differ in their impact on executive control functions [37] so that highly aversive stimuli impact cognitive processes more profoundly than mildly aversive ones [38]. Indeed, several event-related potential (ERP) studies have demonstrated the lesser effect of moderately negative stimuli on response inhibition, in comparison to extremely negative stimuli, hypothesizing that negative events of varying valences are differently processed and differ in their impact on inhibitory functions [38, 39]. In these studies, extremely aversive stimuli were shown to elicit smaller P2 amplitudes, smaller P3 amplitudes and higher N2 amplitudes in comparison to moderately aversive stimuli. Such findings suggest that, in comparison to moderately negative stimuli, extremely negative stimuli facilitated faster stimulus detection, greater intensity of attention and a stronger cognitive control over task-irrelevant information when the aversive stimuli were task-irrelevant [38, 39]. Further evidence suggested that while both women and men are sensitive to the impact of highly negative images, only women are also sensitive to the effect of moderately negative stimuli [40].

### The current study

In the light of the argued differences between both inhibitory sub-functions, a limited number of studies directly compared the performance in GNG and SST, and, to the best of our knowledge, none have used behavioral methods (as opposed to manipulations of neurotransmitters) as differential tools for such an investigation. Thus, the reason for comparing between GNG and SST is twofold. Firstly, we wished to examine whether the manipulation of a behavioral variable (aversive images) would differently impact performance in each task. Such a finding could serve as additional evidence for the necessity to differently conceptualize each inhibitory mechanism, urging researchers to create specific hypotheses regarding the particular inhibitory function(s) they wish to investigate. Secondly, by demonstrating specific effects of aversive stimuli over each inhibitory sub-function, the current results may aid in directing future studies aiming to investigate the modulation of inhibitory sub-functions by emotional manipulations. Furthermore, manipulations which may impact specific inhibitory sub-functions may yield with certain clinical implications (see discussion).

On the current study, we measured the effect of negative stimuli on the performances in both tasks. For this end, we followed the recommendation to combine both tasks into a single paradigm which incorporates go trails, no -go trials (zero-delay inhibitory trials) and stop trials (inhibitory trials in which stop-signal delay is longer than zero) [6, 7]. Examining both inhibitory functions under a single paradigm minimizes potentially confounding effects of comparing between tasks and provides a practical framework for the analysis of inhibitory subtypes [6]. Further, as individuals differences were argued to modulate, at least in part, the effect of emotional material on executive control [41, 42], the operation of a one group within-participants design may aid minimizing the intervening effects of individuals characteristics across groups.

In the light of the diverse effects that valence intensity differences were shown to inflict on executive control functions [37–39], only extremely negative images of low valence rating and which included salient threatening content (e.g. blood, mortal wounds) were selected here. This was undertaken in order to avoid any diverse effects that may take place as a result of valence intensity differences across negative images.

Following past literature of negative emotional stimuli, the current study yielded with three hypotheses. We assumed that the negative stimuli will impair the performance on trials in which the execution of a motor response is required (go trials), thus replicating past findings. Regarding the inhibitory processes, we followed the assumptions that negative stimuli generate enhanced sensory representations of the stop stimulus, consequently leading to an enhanced stopping performance [19, 21, 36, 43] and that the processing of negative stimuli mainly takes place under the control of top-down processes [44–48]. Thus, we expected the negative stimuli to enhance the representation of the stop signs within the top-down inhibitory framework, thereby improving the controlled, top-down inhibitory performance operated by SST. However, we expected the negative stimuli to have little or no impact upon the automatic, bottom-up inhibition, and therefore to have minimal influence upon performance in GNG task.

## Materials and methods

### Participants

54 students (33 women, 21 men, *M*_age_ = 21.7 years, *SD*_age_ = 2.8 years, age range: 19–28 years) of Eötvös Loránd University, Hungary, participated in the current study either voluntarily or for course credit and after signing an informed consent form. All participants had normal or corrected-to-normal vision. The study was approved by the institutional review board of the Faculty of Education and Psychology of Eötvös Loránd University and was conducted in accordance with the Declaration of Helsinki.

### Tools

The experiment was conducted using a Dell PC running PsychoPy, version 1.83.03 [49]. All stimuli were presented at the center of a 17'' LCD Dell monitor. The target stimuli were a circle and a square (2.5 x 2.5cm), similar to the ones appearing on the ''Stop-It'' software [50], which appeared in white on a black background. A white cross (1.5 x 1.5cm) used as a fixation sign. A gray frame (9.5 x 9.5 x 0.5cm) around the target stimuli used as ''go'' sign, and a dashed gray and white frame of the same measurements used as either ''no-go'' sign (i.e., a GNG stop trial in which the stop sign appeared simultaneously with the target stimulus) or ''stop'' sign (i.e., a SST stop trial in which the stop-signal appeared after the appearance of the target stimulus). A feedback sound (750 Hz, 50 dB, 75ms) was heard through earphones (Sennheiser PX-200) whenever an error occurred. 105 negative pictures and 159 neutral pictures (9 x 9cm) were selected based on their valence rating from the Geneva Affective Picture Database (GAPED) [51] and the International Affective Picture System (IAPS) [52], which are standardized sets of images with normative ratings of valence and arousal. As above mentioned, the images were selected according to a strict valence cutoff, which resulted in the two groups of images (negative and neutral) significantly differing both in their levels of valence and arousal. See the Supporting information text for an overview of the selected images and the cutoffs for image selection. As the number of available neutral pictures complying with our strict valence cutoffs was insufficient for the number of required neutral trials, each neutral picture was presented on two random trials throughout the experiment.

### Procedure

**Fig 1:**
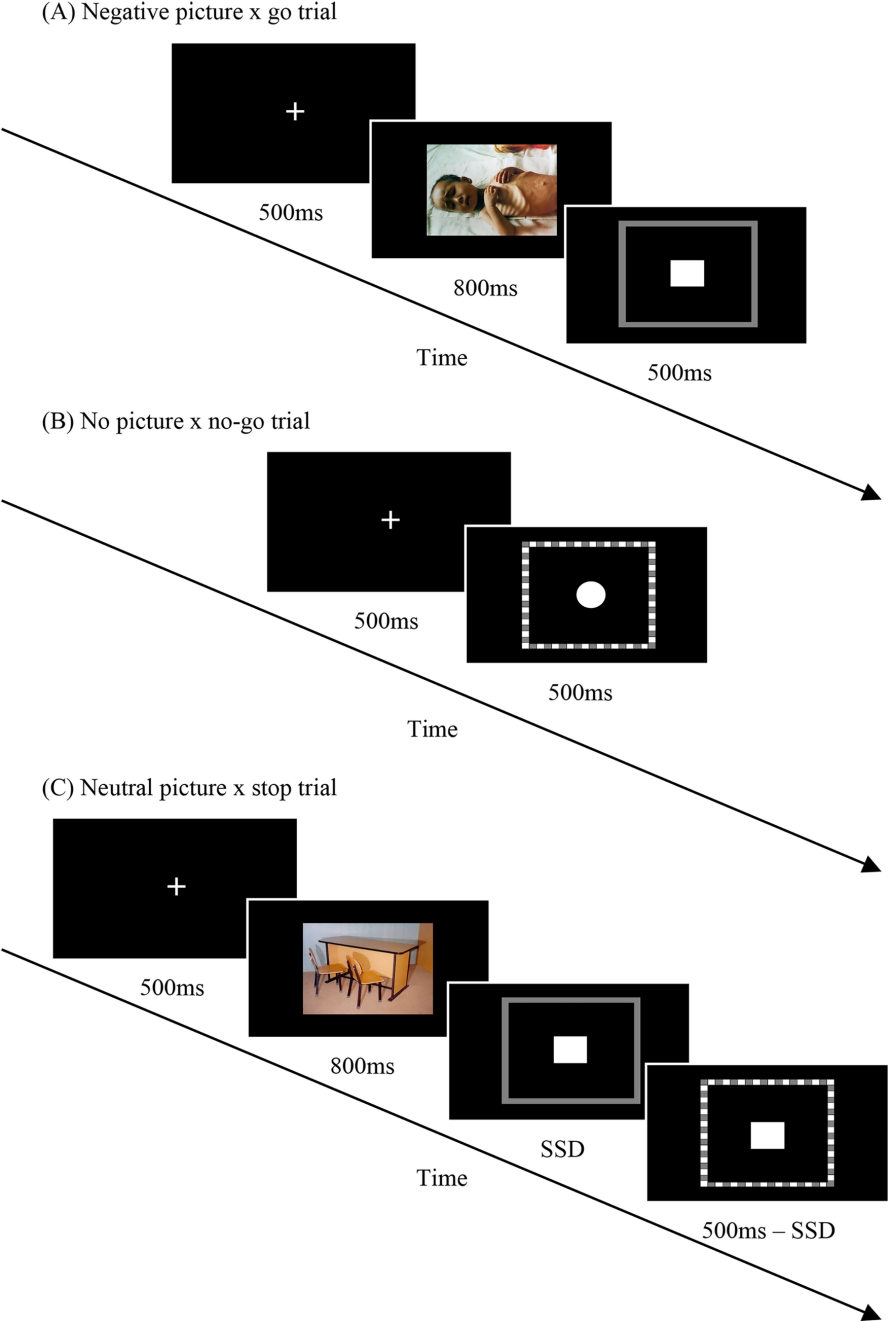
Experimental procedure samples. (A) A negative picture precedes a go trial. (B) A no-go trial appears with no picture prior to it. (C) A neutral picture is followed by a go stimulus, which changes into a stop-signal within the adaptive SSD. SSD = stop-signal delay.

In a within-participants experimental design, each participant was tested individually in front of a monitor in a dimly lighted room after receiving written and verbal instructions. The primary task required participants to press the "A" key whenever a framed square appeared, and to press the "L" key whenever a framed circle appeared. The target stimuli appeared for 500ms. A 500ms fixation cross preceded each trial. On typical inhibitory tasks, the stop trials (or no-go trials) ordinarily constitute around 30% of the total number of trials in the experiment [8, 9]. Thus, on the current experiment, the frame's color changed from gray into dashed gray & white on 30% of the trials, either simultaneously with the appearance of the target stimulus (no-go trial) or within a brief interval (which was set adaptively, see below) after it appeared (stop trial). The frequencies of no-go trials and stop trials were equally distributed. On these trials, the participants were required to inhibit their response and not to press any key. The error sound was activated as a result of pressing the wrong key on go trials, not responding within 500ms on go trials, or pressing any key on no-go trials and stop trials. In between the appearance of the fixation cross and the target stimulus on each trial, a neutral picture, a negative picture or no picture appeared for 800ms (in the event of no picture, the fixation cross was immediately followed by the target stimulus). Each participant underwent all nine combinations of the experimental procedure (the interaction between negative pictures, neutral pictures, and no-pictures with go trials, no-go trials and stop trials). Fig 1 illustrates the experimental procedure.

The experiment started with a practice block of 18 trials followed by 750 experimental trials divided into three blocks of 250 trials each. On each block, the target stimuli appeared in a semi-random order, maintaining the 70–30% proportion of go-stop trials. Additionally, the allocation of go and no-go trials was random within-blocks but amounted to an identical number of trials appearing after each of the picture valence conditions in each block. However, the allocation of stop-trials differed across blocks, with stop-trials following only negative pictures in one block, only neutral pictures on a second block, and only no pictures on a third block. Such allocation of stop-trials was required for the calculation of stop-signal reaction times and their comparison across picture valence conditions (see below). The blocks sequence was distributed equally across participants. Participants were given a two minutes rest in between blocks.

Two core values in SST are stop-signal delay (SSD), which represents the interval between the appearance of the target stimulus and the stop-signal, and stop-signal reaction time (SSRT) which represents the latency of the stop process, i.e. the time it takes one to complete the inhibitory process after the appearance of the stop-signal [9]. While SSD is traditionally manipulated by the researcher, SSRT uses as a measure for one’s inhibitory capacity, with shorter SSRTs indicating superior inhibitory performance. Following the *tracking procedure* for the measurement of SSRT [53], SSD was set dynamically and was adjusted after each stop trial by using a *one-up one-down method*, resulting in an adaptive measurement; the SSD baseline interval on the first trial of each experimental block was 250ms. After successful stopping SSD was increased by 25ms and after unsuccessful stopping SSD was decreased by 25ms. The participants were instructed to respond as fast and as accurately as possible. If the increases and decreases in SSD on each trial are equal in magnitude, the *tracking procedure* should result in an overall of .50 inhibition (and inhibition failure) rate. Thus, the *tracking procedure* compensates for differences between and within participants, controls for difficulty level across participants and results in an approximately similar response/inhibition proportion for different participants, tasks or conditions [53, 54]. A major advantage of the *tracking procedure* is that it allows a simple calculation of SSRT through using the *mean method*, by subtracting the observed mean SSD from the observed mean of the go reaction time (Go-RT) distribution.

### Measurement and statistical analysis plan

Conventionally in GNG, commission errors serve as the index of inhibitory control [5], while SSRT reflects the inhibitory functioning in SST [53, 55]. In order to assess the possible influence of negative stimuli on the functions of response and response inhibition, the following measures were obtained. On go trials, response times and error rates were measured (errors being either lack of response or wrong responses) and compared across picture valence values. On no-go trials, commission error rates (responding to the stimuli, false alarms) were measured and compared across picture valence values to identify any impact of picture valance on inhibitory control in GNG. Additionally, the signal detection measure of *d’* was calculated using the formula:

d’=z(H)–z(FA)

where *z*(H) and *z*(FA) represent the transformation of the hit (correct go trials) and false alarm (commission error) rates to z-scores. The variable *d’* represents a measure of the perceptual sensitivity to different stimulus conditions, indicating how well participants can discriminate and appropriately respond to targets and non-targets, thus further inspecting cognitive control [56, 57]. On stop trials, both commission error rates and the adaptive SSDs on each block were obtained. Using *the mean method* for the assessment of SSRT [53], each participant's mean SSD (resulting from *the tracking procedure*) was calculated for each block and subtracted from their mean Go-RT distribution, resulting in the corresponding SSRT length. As we assumed that the negative stimuli would impact go trials as well (therefore resulting in a different Go-RT distribution than the one that would have emerged without the involvement of the negative stimuli), the mean SSD of each picture valence condition was subtracted from the mean Go-RT for go trials which appeared after no picture trials. SSRTs were then compared across picture valence values (i.e. across blocks). Commission error rates on stop trials were obtained to ensure the .50 inhibition response rate expected of the *tracking procedure*.

A preliminary condition for the calculation of SSRT by using *the mean method* is to evaluate the functionality of *the tracking procedure*. If *the tracking procedure* functioned effectively, the commission error rates on stop trials for each block should be around 50%, indicating that each participant managed to inhibit their response on half of the stop trials in each block. Based on 45 stop trials per block, the 95% confidence interval for proportion around the median was (34.3%, 65.7%) error rate. Only two participants were excluded from the analysis due to error rates outside the confidence interval, indicating that the tracking procedure functioned as expected. Additionally, participants' SSRTs were examined for any negative values. Negative SSRTs indicate that participants did not follow the instruction to respond as quickly as possible to go signals but instead awaited stop-signals in some of the go trials, likely trying to anticipate the stop-signals by slowing down responses [58]. One participant was excluded from the analysis on the grounds of obtaining negative SSRT values. Thus, all reported results are based on the performance of 51 participants. All reported ANOVAs are repeated-measures ANOVAs with picture valence (negative vs. neutral vs. no-picture) as a within-subjects factor. Since each of the inhibitory paradigms has its own unique measure of inhibitory control, separate analyses were conducted for SSRT (inhibitory measure in SST) and commission errors (inhibitory measure in GNG). Additional analyses were conducted for Go-RTs and go error rates (errors of omission and wrong responses), and for the measure of perceptual sensitivity *d’*. Post hoc tests were conducted using the Bonferroni correction. Effect sizes are reported as partial eta squared measures. The Greenhouse-Geisser epsilon correction was applied when necessary to correct a possible lack of sphericity [59].

## Results

### Go trials

**Fig 2:**
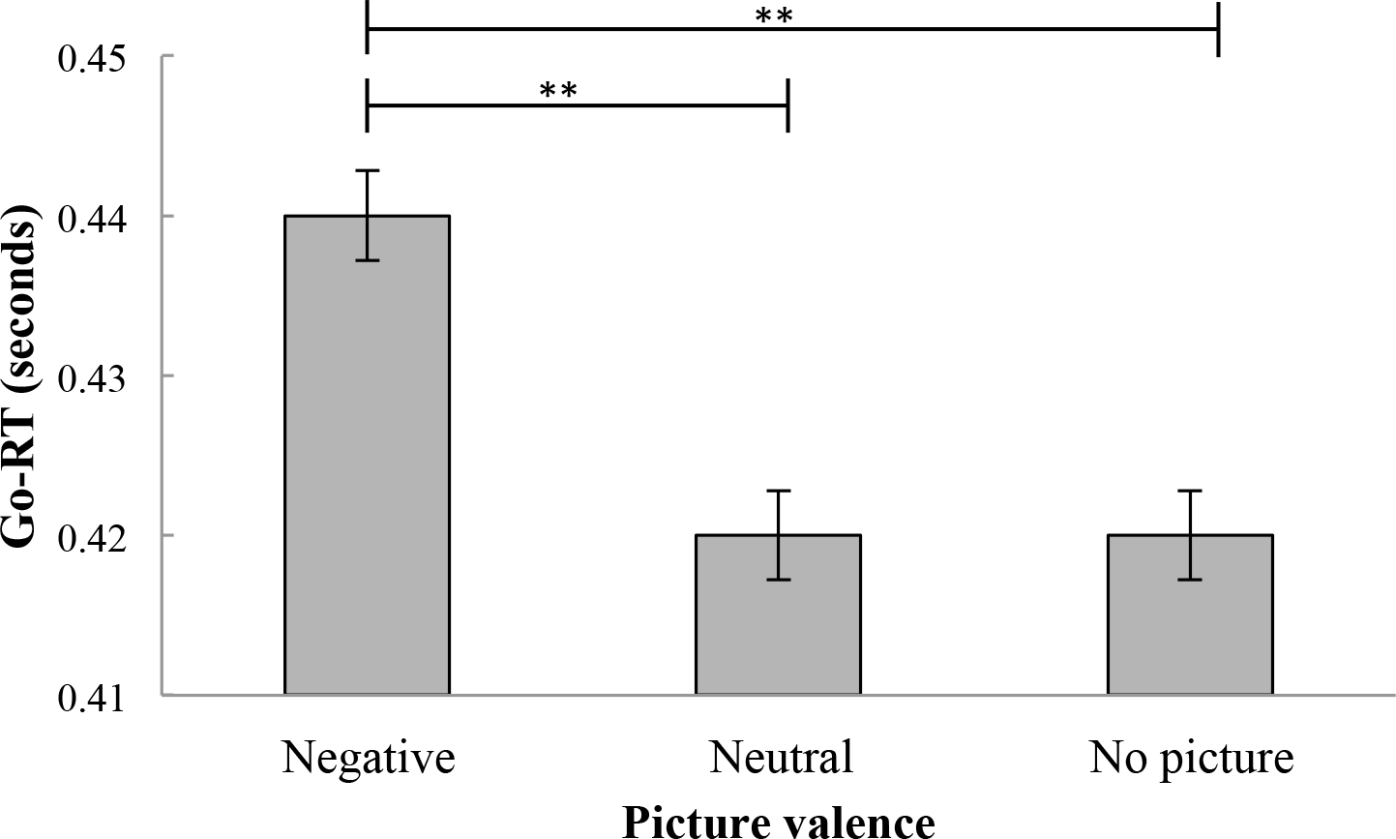
Mean reaction times on go trials (in seconds). Error bars represent standard errors. Response times were longer for stimuli which appeared after negative pictures than for stimuli which appeared after neutral pictures and no pictures. Response times for stimuli which appeared after neutral pictures did not differ from response time for stimuli which appeared after no pictures. Go-RT = go reaction time. **p < .01.

On go trials, response times and error rates were compared across picture valence values. First, an analysis of response times was conducted. Relevant response times means can be found in Table 1. Mean scores for response times were significantly different across picture valence values, *F*(1.42, 70.86) = 21.193, *p* < .01, η_p_^2^ = .30. Post hoc tests revealed that Go-RTs were significantly longer for stimuli which appeared after negative pictures (*M* = .44, *SD* = .02) than for stimuli which appeared both after neutral pictures, (*M* = .42, *SD* = .02), *p* < .001 and after no pictures (*M* = .42, *SD* = .02), *p* < .001. There was no difference between Go-RTs for stimuli which appeared after neutral pictures and no pictures (*p* > .05; see Fig 2).

**Table 1:** Go-RTs and error rates means and standard deviations on go trials as a function of picture valence values.

	Go-RT (seconds)		Error rate (%)	
Picture valence	*M*	*SD*	*M*	*SD*
Negative picture	.44	.02	.41	.16
Neutral picture	.42	.02	.32	.13
No picture	.42	.02	.28	.11

Go-RT, go reaction time.

**Fig 3:**
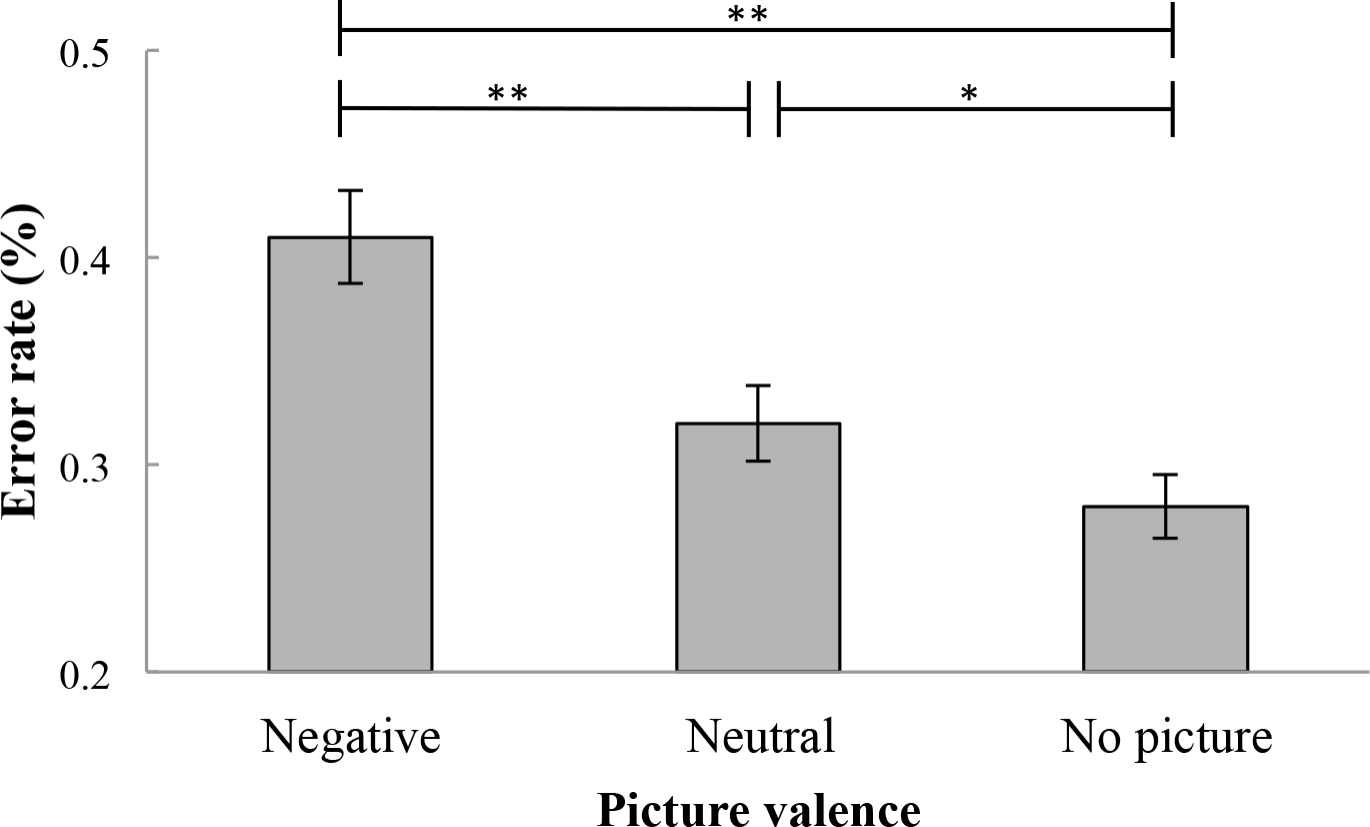
Mean error rates percentage on go trials. Error bars represent standard errors. Higher error rates were found in response to stimuli which appeared after negative pictures than to stimuli which appeared after neutral pictures and no pictures. Higher error rates were also found in response to stimuli which appeared after neutral pictures than to stimuli which appeared after no pictures. *p < .05, **p < .01.

Secondly, an analysis of error rates was conducted. Relevant error rates are presented in Table 1. Error rates were significantly different across picture valence values, *F*(1.51, 75.85) = 41.12, *p* < .001, η_p_^2^ = .45. Post hoc tests revealed that error rates were significantly higher on go trials which appeared after negative pictures (*M* = .41, *SD* = .16) than on go trials which appeared after neutral pictures, (*M* = .32, *SD* = .13), *p* < .001 and after no pictures (*M* = .28, *SD* = .11), *p* < .001. Additionally, error rates were significantly higher on go trials which appeared after neutral pictures in comparison to go trials which appeared after no pictures (*p* < .01; see Fig 3).

### No-go trials

**Fig 4:**
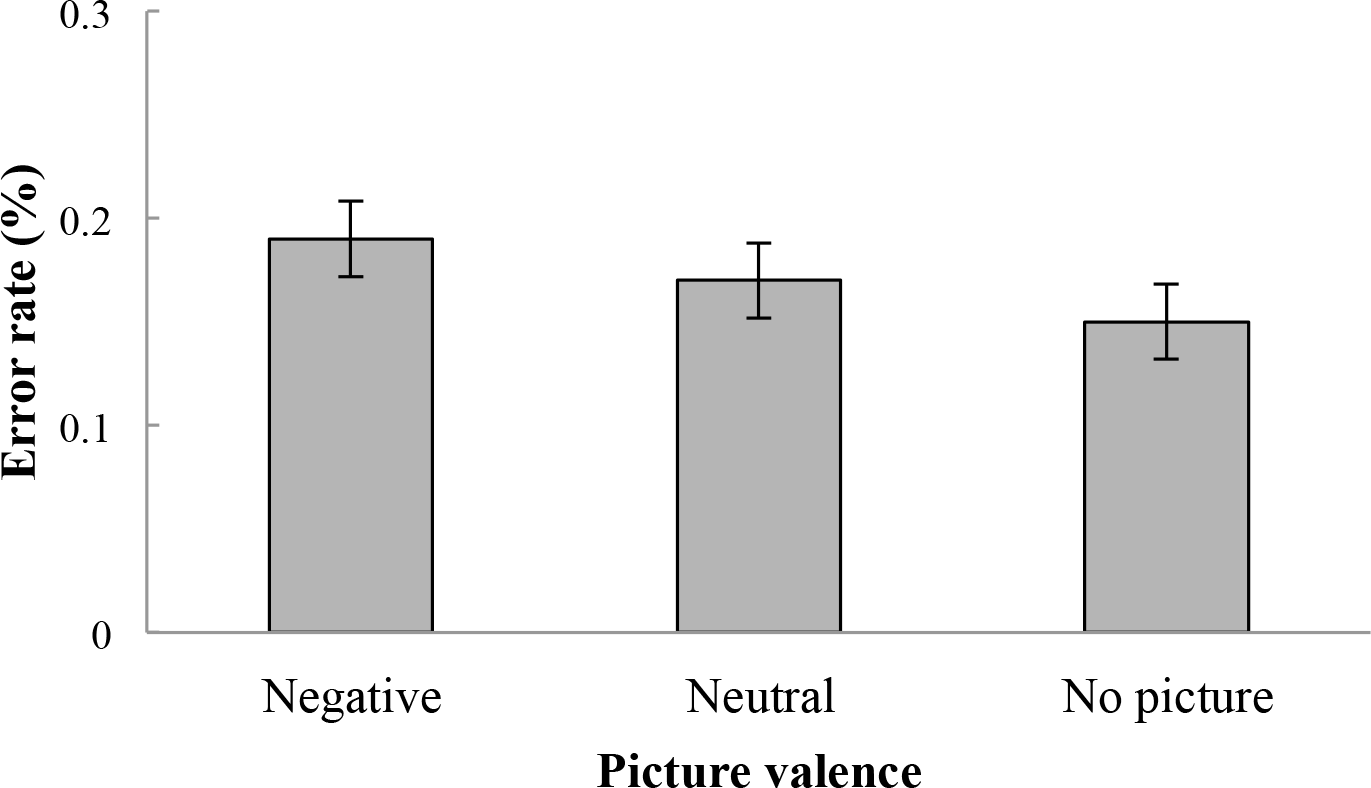
Mean error rates percentage on no-go trials. Error bars represent standard errors. No significant difference in error rate was found in response to stimuli which appeared after negative pictures, neutral pictures, and no pictures.

On no-go trials, an analysis of error rates was conducted. Relevant error rates can be found in Table 2. No significant difference of error rates was found across picture valence values, *F*(1.76, 88.03) = 3.11, *p* > .05. Fig 4 illustrates these results.

**Table 2:** Error rates means and standard deviations on no-go trials as a function of picture valence values.

	Error rate (%)	
Picture valence	*M*	*SD*
Negative picture	.19	.13
Neutral picture	.17	.13
No picture	.15	.13

Additionally, we inspected the possible impact of picture valence on perceptual sensitivity. *d’* measures were significantly different across picture valence values, *F*(2, 100) = 37.04, *p* < .01, η_p_^2^ = .42. Post hoc tests revealed that *d’* scores were significantly lower for trials which appeared after negative pictures (*M* = -.63, *SD* = 1.46) than for trials which appeared after neutral pictures (*M* = .11, *SD* = 1.3), *p* < .01 and after no pictures (*M* = .52, *SD* = 1.31), *p* < .01. Additionally, *d’* scores were significantly lower for trials which appeared after neutral pictures in comparison to trials which appeared after no pictures (*p* < .01).

### Stop trials

**Fig 5:**
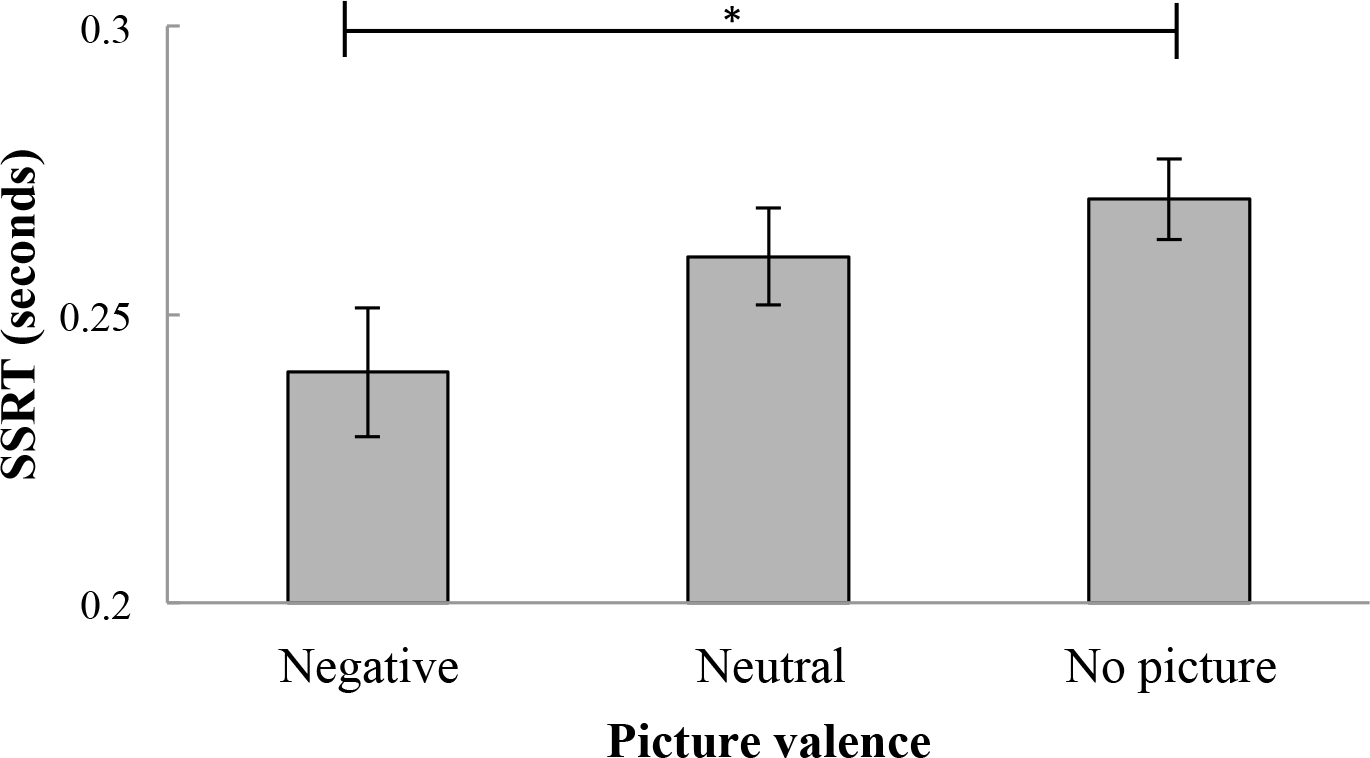
Mean stop-signal reaction times for stop-trials (in seconds). Error bars represent standard errors. SSRTs were shorter for stimuli which appeared after negative pictures than for stimuli which appeared after no pictures. SSRTs for stimuli which appeared after neutral pictures did not differ from SSRTs for stimuli which appeared after negative pictures and no pictures. SSRT = Stop-signal reaction time. *p < .05.

On stop trials, SSRT means were calculated and compared across picture valence values. Relevant SSRT means can be found in Table 3. Mean scores for SSRTs were significantly different across picture valence values, *F*(1.64, 81.79) = 4.88, *p* < .05, η_p_^2^ = .12. Post hoc tests revealed that SSRTs were significantly shorter for stimuli which appeared after negative pictures (*M* = .24, *SD* = .08) than for stimuli which appeared after no pictures (*M* = .27, *SD* = .05), *p* < .05. There was no significant difference between SSRTs for stimuli which appeared after neutral pictures (*M* = .26, *SD* = .06) and SSRTs for stimuli which appeared after either negative pictures or no pictures (*p* > .05), although a marginal significance was found for the difference between SSRTs for stimuli which appeared after neutral pictures and negative pictures (*p* = .083, see Fig 5).

**Table 3:** SSRTs means and standard deviations on stop trials as a function of picture valence values.

	SSRT (seconds)	
Picture valence	*M*	*SD*
Negative picture	.24	.08
Neutral picture	.26	.06
No picture	.27	.05

SSRT, stop-signal reaction time.

## Discussion

In the current study, performances in Go/no-go and Stop-signal paradigms were compared under the influence of negative stimuli. Participants performed a combined task which consisted of go trials, no-go trials, and stop trials, with negative pictures serving as a behavioral tool of interference which was compared against neutral pictures and no pictures conditions. Exposure to negative pictures resulted in longer response times and higher error rates in go trials, in comparison to both neutral pictures and no pictures, suggesting an impairing effect of negative stimuli over performance in go trials. In stop trials, exposure to negative pictures resulted in shorter SSRTs in comparison to no pictures, suggesting an improved inhibitory functioning in SST under the influence of negative stimuli. Last, no significant differences across picture valence values were found in no-go trials, suggesting that performance in GNG was generally unaffected by the exposure to negative stimuli.

The impairing influence of negative stimuli over performance in go trials replicates the findings of past studies, in which negative stimuli were shown to impair executive control and lengthen response times in various cognitive tasks [21, 22, 30, 60, 61], even when presented outside the focus of attention and while task-irrelevant, as in the current study [62–65]. Secondly, the non-significant impact of picture valence on commission errors in no-go trials is supported by past literature as well [28, 29, 31]. As commission error rates are generally considered as the gold standard measure for the assessment of behavioral inhibition in both emotional and classic GNG tasks [5, 29, 56], this finding implies that inhibitory functions in GNG were generally unaffected by negative stimuli. Interestingly, participants’ perceptual sensitivity was in fact modulated by picture valence, with lower *d’* scores indicating difficulties in discriminating and appropriately responding to targets and non-targets (go vs. no-go trials; [57]). However, as the measure of *d’* reflects the ratios of both correct hits and false alarms, and as only one of these measures (correct hits) was significantly modulated by picture valence, this result is unsurprising. Such differences between the inhibitory indicator of commission errors and the perceptual sensitivity measure of *d’* were reported in other studies which made use of both measures [56, 57]. Still, although non-significant, the measure of commission errors in the current study did reflect a general trend of modulation, with negative images yielding with slightly larger commission error rates. Taken together, these findings may reflect a subtle impairing impact of aversive stimuli on inhibitory performance in GNG. Such possible effects could be inspected in future studies which involve measurement tools which are more sensitive to reveal subtle functional differences, such as ERP measures.

Last, the improved inhibitory performance in SST after the exposure to negative stimuli supports the results of former studies in which negative stimuli shortened SSRT and improved inhibitory functioning [35, 36, 66], but is contradictory to those of other studies which reported of longer SSRTs following the exposure to negative pictures [34, 67]. Several researchers [1, 8, 68] argue that such inhibitory tasks are sensitive to task design, emphasizing the necessity for future studies to conceptualize and understand the possible influences of different task designs upon performance. Indeed, the mentioned studies, including the current one, differed in several aspects, including the sensory method of presentation of the stop- signal (visual vs. auditory) and the task design (GNG and SST combined into one task vs. separated into individual tasks). Further comparisons of the influences of negative stimuli in different SST modalities could be executed in future research. However, one important finding that emerges from the described body of studies is that negative stimuli may impact the inhibitory process taking place in SST (even if the direction of such impact is still debated), but not the one taking place in GNG. These findings may yield with relevant clinical implications (see below). Additionally, the difference between SSRTs following negative pictures and neutral pictures appeared in trend, with negative pictures resulting in shorter SSRTs than neutral pictures, but failed to reach significance. Aichert et al. [58] noted that studies with sample sizes similar to the one used in the current study are only moderately powered to detect associations of small magnitude, and it is possible that this effect would turn significant on a larger sample size.

In the current study, negative stimuli were shown to affect differently the performance in GNG and SST. Following Schachar et al.'s typologies [7], these results depict an interesting picture after which a behavioral tool was shown to improve action cancellation performance, but had no impact upon action restraint. One possible explanation for these findings alludes to the involvement of different neurotransmitters in both tasks. As above discussed, noradrenaline was shown to affect performance mainly in SST, but not in GNG [6, 69, 70]. Since negative stimuli were shown to increase levels of noradrenaline [24, 25], it could be hypothesized that the effects found in the current study are the outcome of such noradrenergic activity which was evoked by the exposure to negative stimuli and affected performance in SST, but not in GNG. Although this reasoning is in line with the findings after which an improvement in SST performance followed the application of noradrenergic agonist [71], such conclusions are beyond the scope of the current study and should be investigated under experimental designs which involve electrophysiological and neurological measures in addition to behavioral ones.

A supplementary explanation for the findings is the one brought above, after which top-down inhibition, but not bottom-up inhibition, was most affected by the negative stimuli. Such an effect may have been caused by the competition of the negative stimuli over of top-down processing capacity resources, as earlier suggested [43, 47, 72]. If such a competition over processing resources indeed took place within the top-down inhibitory process, it would be expected to lengthen the time it took participants to resolve the conflict between response and inhibition taking place in stop trials, thus resulting in higher chances of avoiding from (or failing to) executing a response to the target stimuli. Such process would account for the higher chances of inhibiting response in stop trials that followed the exposure to negative stimuli.

The accumulating findings from neurological, neurochemical and behavioral studies brought here implicate of a core difference between GNG and SST, which possibly measure two separate cognitive mechanisms. As a continuation of the current approach, future studies could compare the possible impact of emotional stimuli over the performance in other inhibitory tasks, which are considered to be even more closely related to one another by measurement of automatic inhibition solely. Such a comparison, for example, could include a GNG task and a two-choice oddball task, which is similar to the classic GNG task but requires motor responses to both go and no-go trials, thus obviating any possible confounding effects that could arise from the fact that go trials involves motor responses whereas no-go trials do not [73, 74]. Another possible comparison could involve the influence of emotional stimuli on both SST and oddball task. Such investigations could potentially support our better understanding of the differences and similarities between tasks, and the potential modulating effect of emotional interventions upon each.

### Implications for treatment in inhibitory impaired conditions

Impaired response inhibition was found in various clinical populations diagnosed with bipolar disorder [75], substance abuse [76, 77], adolescence smoking [78] borderline personality disorder [79, 80], schizophrenia [81, 82], and Attention Deficit Hyperactivity Disorder (ADHD) [83–86]. In a recent study of combat veterans with and without post-traumatic stress disorder (PTSD; [87]), the authors found that higher levels of PTSD and depressive symptoms were associated with higher error rates in a GNG task. Since cognitive impairments could hinder the effectiveness of standard PTSD therapies which are based upon cognitive reappraisal and disengagement from traumatic stimuli (see [88] for a review), the authors recommend integrating treatments that strengthen executive functions within traditional PTSD treatments. Additionally, Chambers et al. [13] note that inhibition-related regions whose activity increased with practice are the same areas that were shown to differentiate between ‘good’ and ‘poor’ performers. The authors consider these findings promising from a clinical perspective, demonstrating that there is plasticity in the brain centers that underlie a clinically important inhibitory function.

Interestingly, recent studies have demonstrated a possible use of GNG task in influencing maladaptive inhibitory-related behaviors. Makin use of a modified GNG task [89], a significant reduction in weekly alcohol intake was demonstrated in a group of heavy drinkers when alcohol-related stimuli were consistently paired with the no-go condition and a significant increase in weekly alcohol intake when alcohol-related stimuli were paired with the go condition. Using a similar paradigm, other studies managed to reduce the impulsive processes of eating behavior [90–94], to demonstrate long-term effects of weight reduction [95], and to diminish the attractiveness of sexually appealing images [96]. See [97] for a review of the cognitive mechanisms operated by these training methods.

However, several meta-analytic studies which were conducted to determine the effects of inhibitory control training over the reduction of harmful behaviors [98–100] have recently found that GNG training, rather than SST training, most influenced participants’ health behavior. Similar results were reported by a study in which the impact of both tasks on food consumption was compared [101]. These findings imply that by redesigning cognitive training GNG tasks we may be able to strengthen individuals’ automatic inhibitory capacity. Importantly, the interaction effect found in the current study supports the notion that negative emotional stimuli may boost, at least temporarily, controlled forms of inhibition. Indeed, past studies have claimed that different types of practice are expected to influence performance in either GNG or SST [16, 102], and suggested two different types of inhibitory training methods to improve deficits in inhibitory control; a bottom-up training, based on GNG paradigm, and a top-down training, based on SST (see [103] for a short review). Such differentiation may prove crucial in the light of the possible inhibitory impairment differences across pathologies. For example, two meta-analytic studies indicated impairments in GNG performance, but not in SST performance in patients diagnosed with bipolar disorder, autism, and Tourette syndrome, and a diverse pattern of impairment for reading disorder. Other disorders, such as schizophrenia, were shown to differ in magnitude of impairment across tasks, with greater impairment in of action cancellation than of action restraint [83, 104]. Thus, when planning an inhibitory-based training intervention, it is essential to match a suitable training paradigm for the impaired inhibitory sub-type. The findings of the current study may aid in the development of interventions aimed at ameliorating participants’ controlled inhibitory functions.

### Limitations

As the pictures in IAPS and GAPED picture databases are rated on the factors of valence and level of arousal, and since both variables are correlated so that the highly negative pictures chosen for the current study are also highly arousing and the neutral pictures are low on their level of arousal, the current results could be explained as caused by level of arousal experienced by participants, and not by the pictures valence. Indeed, several studies [36, 67] suggested that the level of arousal evoked by the stimuli, and not the stimuli's valence, was the cause of impact. The separation between these two variables was not applicable to the current study's design and could be further investigated in future research. Nonetheless, and apart from the theoretical importance of such differentiation, the impact of the negative stimuli on SST functioning, but not on GNG functioning, preserves its significance regardless of the unique variable which may have caused the effect.

A second potential limitation is related to the repetition of each neutral picture twice throughout the experiment, an issue which may have resulted in a certain amount of habituation to the neutral images. However, we believe such outcome is unlikely as the effects of habituation were previously shown to be minimal for neutral images, even when images were repeatedly presented [105, 106]. Furthermore, in all cases where negative stimuli were shown to impact performance in the current study (i.e., Go-RTs, Go omission errors and SSRTs), performance was shown to differ significantly in comparison to the no-picture condition, thus highlighting the impact of the negative stimuli regardless of the responses to the neutral ones.

Last, as above mentioned, only extremely aversive images were selected for the current study. Since moderately aversive stimuli were shown to result in milder impact upon inhibitory functioning [38, 39], the implications of the current study should be limited to the impact of highly negative stimuli. Such differentiations are specifically important as certain behavioral measures may be less sensitive than physiological ones to detect subtle effects of stimuli upon inhibitory functioning [26, 38, 73]. Future studies should further inspect the effect of diverse levels of negative valence upon inhibitory capacity while incorporating the use of physiological measures in addition to behavioral ones.

## Conclusion

In the current study, performance in Go/no-go and Stop-signal tasks was shown to be differently affected by the exposure to negative stimuli among university students. While exposure to negative stimuli impaired performance in go trials and improved inhibitory functions in Stop-signal task, inhibitory performance in Go/no-go task was generally unaffected. These findings support the conceptual differentiation between two subtypes of inhibitory functions, urging researchers to hypothesize upon accurate inhibitory typologies and pair each to its suitable measure, thus forwarding a thorough understanding of the complex structures of inhibitory control. Further, these findings illustrate a possible intervention to impact top-down inhibitory control. As the source of inhibitory impairment (automatic vs. controlled) may differ across clinical conditions, inhibitory-training interventions which are tailored to the subject of impairment may prove beneficial for individuals struggling with inhibitory deficiencies.

## Supporting information

S1 Table Valence and arousal means for images selected out of GAPED image bank. (DOCX)

S2 Table Valence and arousal means for images selected out of IAPS image bank. (DOCX)

S3 Table An overview of the selected images. (DOCX)

## References

[1] Bari A, Robbins TW. Inhibition and impulsivity: behavioral and neural basis of response control. Prog Neurobiol. 2013;108:44–79. doi: 10.1016/j.pneurobio.2013.06.005 23856628 10.1016/j.pneurobio.2013.06.005

[2] Goldstein M, Brendel G, Tuescher O, Pan H, Epstein J, Beutel M, et al Neural substrates of the interaction of emotional stimulus processing and motor inhibitory control: an emotional linguistic go/no-go fMRI study. Neuroimage. 2007;36(3):1026–40. doi: 10.1016/j.neuroimage.2007.01.056 17509899 10.1016/j.neuroimage.2007.01.056

[3] Aron AR, Robbins TW, Poldrack RA. Inhibition and the right inferior frontal cortex. Trends Cogn Sci. 2004;8(4):170–7. doi: 10.1016/j.tics.2004.02.010 15050513 10.1016/j.tics.2004.02.010

[4] Friedman NP, Miyake A. The relations among inhibition and interference control functions: a latent-variable analysis. J Exp Psychol Gen. 2004;133(1):101–35. doi: 10.1037/0096-3445.133.1.101 14979754 10.1037/0096-3445.133.1.101

[5] Aron AR, Poldrack RA. The cognitive neuroscience of response inhibition: relevance for genetic research in attention-deficit/hyperactivity disorder. Biol Psychiatry. 2005;57(11):1285–92. doi: 10.1016/j.biopsych.2004.10.026 15950000 10.1016/j.biopsych.2004.10.026

[6] Eagle DM, Bari A, Robbins TW. The neuropsychopharmacology of action inhibition: cross-species translation of the stop-signal and go/no-go tasks. Psychopharmacology (Berl). 2008;199(3):439–56. 18542931 10.1007/s00213-008-1127-6

[7] Schachar R, Logan GD, Robaey P, Chen S, Ickowicz A, Barr C. Restraint and cancellation: multiple inhibition deficits in attention deficit hyperactivity disorder. J Abnorm Child Psychol. 2007;35(2):229–38. doi: 10.1007/s10802-006-9075-2 17351752 10.1007/s10802-006-9075-2

[8] Simmonds DJ, Pekar JJ, Mostofsky SH. Meta-analysis of Go/No-go tasks demonstrating that fMRI activation associated with response inhibition is task-dependent. Neuropsychologia. 2008;46(1):224–32. doi: 10.1016/j.neuropsychologia.2007.07.015 17850833 10.1016/j.neuropsychologia.2007.07.015 PMC2327217

[9] Verbruggen F, Logan GD. Response inhibition in the stop-signal paradigm. Trends Cogn Sci. 2008;12(11):418–24. doi: 10.1016/j.tics.2008.07.005 18799345 10.1016/j.tics.2008.07.005 PMC2709177

[10] López-Caneda E, Rodríguez Holguín S, Cadaveira F, Corral M, Doallo S. Impact of alcohol use on inhibitory control (and vice versa) during adolescence and young adulthood: a review. Alcohol Alcohol. 2014;49(2):173–81. doi: 10.1093/alcalc/agt168 24243684 10.1093/alcalc/agt168

[11] Rubia K, Russell T, Overmeyer S, Brammer MJ, Bullmore ET, Sharma T, et al Mapping motor inhibition: conjunctive brain activations across different versions of go/no-go and stop tasks. Neuroimage. 2001;13(2):250–61. doi: 10.1006/nimg.2000.0685 11162266 10.1006/nimg.2000.0685

[12] Swick D, Ashley V, Turken U. Are the neural correlates of stopping and not going identical? Quantitative meta-analysis of two response inhibition tasks. Neuroimage. 2011;56(3):1655–65. doi: 10.1016/j.neuroimage.2011.02.070 21376819 10.1016/j.neuroimage.2011.02.070

[13] Chambers CD, Garavan H, Bellgrove MA. Insights into the neural basis of response inhibition from cognitive and clinical neuroscience. Neurosci Biobehav Rev. 2009;33(5):631–46. doi: 10.1016/j.neubiorev.2008.08.016 18835296 10.1016/j.neubiorev.2008.08.016

[14] Eagle DM, Lehmann O, Theobald DE, Pena Y, Zakaria R, Ghosh R, et al Serotonin depletion impairs waiting but not stop-signal reaction time in rats: implications for theories of the role of 5-HT in behavioral inhibition. Neuropsychopharmacology. 2009;34(5):1311–21. doi: 10.1038/npp.2008.202 19005464 10.1038/npp.2008.202

[15] Rubia K, Smith A, Taylor E. Performance of children with attention deficit hyperactivity disorder (ADHD) on a test battery of impulsiveness. Child Neuropsychol. 2007;13(3):276–304. doi: 10.1080/09297040600770761 17453834 10.1080/09297040600770761

[16] Verbruggen F, Logan GD. Automatic and controlled response inhibition: associative learning in the go/no-go and stop-signal paradigms. J Exp Psychol Gen. 2008;137(4):649–72. doi: 10.1037/a0013170 18999358 10.1037/a0013170 PMC2597400

[17] Aron AR. From reactive to proactive and selective control: developing a richer model for stopping inappropriate responses. Biol Psychiatry. 2011;69(12):e55–68. doi: 10.1016/j.biopsych.2010.07.024 20932513 10.1016/j.biopsych.2010.07.024 PMC3039712

[18] Chajut E, Mama Y, Levy L, Algom D. Avoiding the approach trap: a response bias theory of the emotional Stroop effect. J Exp Psychol Learn Mem Cogn. 2010;36(6):1567–72. doi: 10.1037/a0020710 20854007 10.1037/a0020710

[19] Fox E, Russo R, Bowles R, Dutton K. Do threatening stimuli draw or hold visual attention in subclinical anxiety? J Exp Psychol Gen. 2001;130(4):681–700. 11757875 PMC1924776

[20] Schimmack U, Derryberry D. Attentional interference effects of emotional pictures: threat, negativity, or arousal? Emotion. 2005;5(1):55–66. doi: 10.1037/1528-3542.5.1.55 15755219 10.1037/1528-3542.5.1.55

[21] Algom D, Chajut E, Lev S. A rational look at the emotional stroop phenomenon: a generic slowdown, not a stroop effect. J Exp Psychol Gen. 2004;133(3):323–38. doi: 10.1037/0096-3445.133.3.323 15355142 10.1037/0096-3445.133.3.323

[22] Estes Z, Adelman JS. Automatic vigilance for negative words is categorical and general.: Emotion; 2008. 10.1037/1528-3542.8.4.441 18729575

[23] Koster EH, Crombez G, Verschuere B, De Houwer J. Selective attention to threat in the dot probe paradigm: differentiating vigilance and difficulty to disengage. Behav Res Ther. 2004;42(10):1183–92. doi: 10.1016/j.brat.2003.08.001 15350857 10.1016/j.brat.2003.08.001

[24] Codispoti M, Gerra G, Montebarocci O, Zaimovic A, Raggi MA, Baldaro B. Emotional perception and neuroendocrine changes. Psychophysiology. 2003;40(6):863–8. 14986839 10.1111/1469-8986.00104

[25] McGaugh JL. Make mild moments memorable: add a little arousal. Trends Cogn Sci. 2006;10(8):345–7. doi: 10.1016/j.tics.2006.06.001 16793325 10.1016/j.tics.2006.06.001

[26] Elliott R, Rubinsztein JS, Sahakian BJ, Dolan RJ. Selective attention to emotional stimuli in a verbal go/no‐go task: an fMRI study. Neuroreport. 2000;11(8):1739–44. 10852235 10.1097/00001756-200006050-00028

[27] Shafritz KM, Collins SH, Blumberg HP. The interaction of emotional and cognitive neural systems in emotionally guided response inhibition. Neuroimage. 2006;31(1):468–75. doi: 10.1016/j.neuroimage.2005.11.053 16480897 10.1016/j.neuroimage.2005.11.053

[28] Chamberlain SR, Fineberg NA, Blackwell AD, Clark L, Robbins TW, Sahakian BJ. A neuropsychological comparison of obsessive-compulsive disorder and trichotillomania. Neuropsychologia. 2007;45(4):654–62. doi: 10.1016/j.neuropsychologia.2006.07.016 17005210 10.1016/j.neuropsychologia.2006.07.016

[29] Gole M, Köchel A, Schäfer A, Schienle A. Threat engagement, disengagement, and sensitivity bias in worry-prone individuals as measured by an emotional go/no-go task. J Behav Ther Exp Psychiatry. 2012;43(1):532–9. doi: 10.1016/j.jbtep.2011.07.002 21819812 10.1016/j.jbtep.2011.07.002

[30] Hare TA, Tottenham N, Davidson MC, Glover GH, Casey BJ. Contributions of amygdala and striatal activity in emotion regulation. Biol Psychiatry. 2005;57(6):624–32. doi: 10.1016/j.biopsych.2004.12.038 15780849 10.1016/j.biopsych.2004.12.038

[31] Silbersweig D, Clarkin JF, Goldstein M, Kernberg OF, Tuescher O, Levy KN, et al Failure of frontolimbic inhibitory function in the context of negative emotion in borderline personality disorder. Am J Psychiatry. 2007;164(12):1832–41. doi: 10.1176/appi.ajp.2007.06010126 18056238 10.1176/appi.ajp.2007.06010126

[32] Albert J, López-Martín S, Carretié L. Emotional context modulates response inhibition: neural and behavioral data. Neuroimage. 2010;49(1):914–21. doi: 10.1016/j.neuroimage.2009.08.045 19716425 10.1016/j.neuroimage.2009.08.045

[33] De Houwer J, Tibboel H. Stop what you are not doing! Emotional pictures interfere with the task not to respond. Psychon Bull Rev. 2010;17(5):699–703. doi: 10.3758/PBR.17.5.699 21037169 10.3758/PBR.17.5.699

[34] Kalanthroff E, Cohen N, Henik A. Stop feeling: inhibition of emotional interference following stop-signal trials. Front Hum Neurosci. 2013;7:78 doi: 10.3389/fnhum.2013.00078 23503817 10.3389/fnhum.2013.00078 PMC3596782

[35] Pawliczek CM, Derntl B, Kellermann T, Kohn N, Gur RC, Habel U. Inhibitory control and trait aggression: neural and behavioral insights using the emotional stop signal task. Neuroimage.;79:264–74.2013 doi: 10.1016/j.neuroimage.2013.04.104 23660028 10.1016/j.neuroimage.2013.04.104

[36] Pessoa L, Padmala S, Kenzer A, Bauer A. Interactions between cognition and emotion during response inhibition. Emotion. 2012;12(1):192–7. doi: 10.1037/a0024109 21787074 10.1037/a0024109 PMC3208031

[37] Pessoa L. How do emotion and motivation direct executive control?. Trends in cognitive sciences. 2009; 13(4):160–6. doi: 10.1016/j.tics.2009.01.006 19285913 10.1016/j.tics.2009.01.006 PMC2773442

[38] Yuan J, Meng X, Yang J, Yao G, Hu L, Yuan H. The valence strength of unpleasant emotion modulates brain processing of behavioral inhibitory control: Neural correlates. Biological psychology. 2012; 89(1):240–51. doi: 10.1016/j.biopsycho.2011.10.015 22056697 10.1016/j.biopsycho.2011.10.015

[39] Yuan J, Zhang Q, Chen A, Li H, Wang Q, Zhuang Z, et al Are we sensitive to valence differences in emotionally negative stimuli? Electrophysiological evidence from an ERP study. Neuropsychologia. 2007; 45(12):2764–71. doi: 10.1016/j.neuropsychologia.2007.04.018 17548095 10.1016/j.neuropsychologia.2007.04.018

[40] Li H, Yuan J, Lin C. The neural mechanism underlying the female advantage in identifying negative emotions: An event-related potential study. Neuroimage. 2008; 40(4):1921–9. doi: 10.1016/j.neuroimage.2008.01.033 18343686 10.1016/j.neuroimage.2008.01.033

[41] Okon-Singer H, Lichtenstein-Vidne L, Cohen N. Dynamic modulation of emotional processing. . Biological psychology. 2013; 92(3):480–91. doi: 10.1016/j.biopsycho.2012.05.010 22676964 10.1016/j.biopsycho.2012.05.010

[42] Cohen N, Henik A. Do irrelevant emotional stimuli impair or improve executive control?. Frontiers in Integrative Neuroscience. 2012;6. 10.3389/fnint.2012.00033 PMC3376948 22719722

[43] Vuilleumier P. How brains beware: neural mechanisms of emotional attention. Trends Cogn Sci. 2005;9(12):585–94. doi: 10.1016/j.tics.2005.10.011 16289871 10.1016/j.tics.2005.10.011

[44] Hahn S, Gronlund SD. Top-down guidance in visual search for facial expressions. Psychon Bull Rev. 2007;14(1):159–65. 17546747 10.3758/bf03194044

[45] Mohanty A, Egner T, Monti JM, Mesulam MM. Search for a threatening target triggers limbic guidance of spatial attention. J Neurosci. 2009;29(34):10563–72. doi: 10.1523/JNEUROSCI.1170-09.2009 19710309 10.1523/JNEUROSCI.1170-09.2009 PMC4348011

[46] Mohanty A, Sussman TJ. Top-down modulation of attention by emotion. Front Hum Neurosci. 2013;7:102 doi: 10.3389/fnhum.2013.00102 23554590 10.3389/fnhum.2013.00102 PMC3612596

[47] Pessoa L, Kastner S, Ungerleider LG. Attentional control of the processing of neural and emotional stimuli. Brain Res Cogn Brain Res. 2002;15(1):31–45. 12433381 10.1016/s0926-6410(02)00214-8

[48] Pessoa L. On the relationship between emotion and cognition. Nat Rev Neurosci. 2008; 9(2):148–58. doi: 10.1038/nrn2317 18209732 10.1038/nrn2317

[49] Peirce JW. PsychoPy—Psychophysics software in Python. J Neurosci Methods. 2007; 162(1–2):8–13. doi: 10.1016/j.jneumeth.2006.11.017 17254636 10.1016/j.jneumeth.2006.11.017 PMC2018741

[50] Verbruggen F, Logan GD, Stevens MA. STOP-IT: Windows executable software for the stop-signal paradigm. Behav Res Methods. 2008;40(2):479–83. 18522058 10.3758/brm.40.2.479

[51] Dan-Glauser ES, Scherer KR. The Geneva affective picture database (GAPED): a new 730-picture database focusing on valence and normative significance. Behav Res Methods. 2011;43(2):468–77. doi: 10.3758/s13428-011-0064-1 21431997 10.3758/s13428-011-0064-1

[52] Lang PJ, Bradley MM, Cuthbert BN. International affective picture system (IAPS): Affective ratings of pictures and instruction manual 2008 University of Florida, Gainesville, FL.

[53] Verbruggen F, Logan GD. Models of response inhibition in the stop-signal and stop-change paradigms. Neurosci Biobehav Rev. 2009;33(5):647–61. doi: 10.1016/j.neubiorev.2008.08.014 18822313 10.1016/j.neubiorev.2008.08.014 PMC2696813

[54] Congdon E, Mumford JA, Cohen JR, Galvan A, Canli T, Poldrack RA. Measurement and reliability of response inhibition. Front Psychol. 2012;3:37 doi: 10.3389/fpsyg.2012.00037 22363308 10.3389/fpsyg.2012.00037 PMC3283117

[55] Hamilton KR, Littlefield AK, Anastasio NC, Cunningham KA, Fink LH, Wing VC, et al Rapid-response impulsivity: definitions, measurement issues, and clinical implications. Personality Disorders: Theory, Research, and Treatment. 2015;6(2):168. 10.1037/per0000100 PMC4476624 25867840

[56] Schulz KP, Fan J, Magidina O, Marks DJ, Hahn B, Halperin JM. Does the emotional go/no-go task really measure behavioral inhibition?: Convergence with measures on a non-emotional analog. Archives of Clinical Neuropsychology. 2007;22(2):151–60. doi: 10.1016/j.acn.2006.12.001 17207962 10.1016/j.acn.2006.12.001 PMC2562664

[57] Singh MK, Chang KD, Mazaika P, Garrett A, Adleman N, Kelley R, et al Neural correlates of response inhibition in pediatric bipolar disorder. Journal of child and adolescent psychopharmacology. 2010;20(1):15–24. doi: 10.1089/cap.2009.0004 20166792 10.1089/cap.2009.0004 PMC2835388

[58] Aichert DS, Wöstmann NM, Costa A, Macare C, Wenig JR, Möller HJ, et al Associations between trait impulsivity and prepotent response inhibition. J Clin Exp Neuropsychol. 2012;34(10):1016–32. doi: 10.1080/13803395.2012.706261 22888795 10.1080/13803395.2012.706261

[59] Geisser S, Greenhouse SW. An extension of box's results on the use of the F distribution in multivariate analysis. The Annals of Mathematical Statistics. 1958;29(3):885–91. 1958.

[60] Dennis TA, Chen CC, McCandliss BD. Threat-related attentional biases: an analysis of three attention systems. Depress Anxiety. 2008;25(6):E1–E10. doi: 10.1002/da.20308 17565734 10.1002/da.20308 PMC2662699

[61] Estes Z, Verges M. Freeze or flee? Negative stimuli elicit selective responding. Cognition. 2008;108(2):557–65. doi: 10.1016/j.cognition.2008.03.003 18433742 10.1016/j.cognition.2008.03.003

[62] Meng X, Yuan J, Li H. Automatic processing of valence differences in emotionally negative stimuli: evidence from an ERP study. Neuroscience letters. 2009; 464(3):228–32. doi: 10.1016/j.neulet.2009.08.064 19720111 10.1016/j.neulet.2009.08.064

[63] Chajut E, Schupak A, Algom D. Emotional dilution of the Stroop effect: A new tool for assessing attention under emotion. Emotion. 2010; 10(6):944 doi: 10.1037/a0020172 21058847 10.1037/a0020172

[64] Vuilleumier P, Armony JL, Driver J, Dolan RJ. Effects of attention and emotion on face processing in the human brain: an event-related fMRI study. Neuron. 2001;30(3):829–41. 11430815 10.1016/s0896-6273(01)00328-2

[65] Chen M, Bargh JA. Consequences of automatic evaluation: Immediate behavioral predispositions to approach or avoid the stimulus. Personality and social psychology bulletin. 1999;25(2):215–24.

[66] Senderecka M. Threatening visual stimuli influence response inhibition and error monitoring: An event-related potential study. Biological psychology. 2016;113:24–36. doi: 10.1016/j.biopsycho.2015.11.003 26599814 10.1016/j.biopsycho.2015.11.003

[67] Verbruggen F, De Houwer J. Do emotional stimuli interfere with response inhibition? Evidence from the stop-signal paradigm. Cognition and Emotion; 2007. 10.1080/02699931.2021.1979475 34556000

[68] Sagaspe P, Schwartz S, Vuilleumier P. Fear and stop: a role for the amygdala in motor inhibition by emotional signals. Neuroimage. 2011;55(4):1825–35. doi: 10.1016/j.neuroimage.2011.01.027 21272655 10.1016/j.neuroimage.2011.01.027

[69] Chamberlain SR, Müller U, Blackwell AD, Clark L, Robbins TW, Sahakian BJ. Neurochemical modulation of response inhibition and probabilistic learning in humans. Science. 2006;311(5762):861–3. doi: 10.1126/science.1121218 16469930 10.1126/science.1121218 PMC1867315

[70] Dalley JW, Everitt BJ, Robbins TW. Impulsivity, compulsivity, and top-down cognitive control. Neuron. 2011;69(4):680–94. doi: 10.1016/j.neuron.2011.01.020 21338879 10.1016/j.neuron.2011.01.020

[71] Robbins TW, Arnsten AF. The neuropsychopharmacology of fronto-executive function: monoaminergic modulation. Annu Rev Neurosci. 2009;32:267–87. doi: 10.1146/annurev.neuro.051508.135535 19555290 10.1146/annurev.neuro.051508.135535 PMC2863127

[72] Padmala S, Bauer A, Pessoa L. Negative emotion impairs conflict-driven executive control. Front Psychol. 2011;2:192 doi: 10.3389/fpsyg.2011.00192 21886635 10.3389/fpsyg.2011.00192 PMC3154405

[73] Wang Y, Yang J, Yuan J, Fu A, Meng X, Li H. The impact of emotion valence on brain processing of behavioral inhibitory control: Spatiotemporal dynamics. Neuroscience letters. 2011;502(2):112–6. doi: 10.1016/j.neulet.2011.07.039 21827832 10.1016/j.neulet.2011.07.039

[74] Yuan J, He Y, Qinglin Z, Chen A, Li H. Gender differences in behavioral inhibitory control: ERP evidence from a two‐choice oddball task. Psychophysiology. 2008;45(6):986–93. doi: 10.1111/j.1469-8986.2008.00693.x 18778319 10.1111/j.1469-8986.2008.00693.x

[75] Fleck DE, Kotwal R, Eliassen JC, Lamy M, Delbello MP, Adler CM, et al Preliminary evidence for increased frontosubcortical activation on a motor impulsivity task in mixed episode bipolar disorder. J Affect Disord. 2011;133(1–2):333–9. doi: 10.1016/j.jad.2011.03.053 21546091 10.1016/j.jad.2011.03.053 PMC3156269

[76] Monterosso JR, Aron AR, Cordova X, Xu J, London ED. Deficits in response inhibition associated with chronic methamphetamine abuse. Drug Alcohol Depend. 2005;79(2):273–7. doi: 10.1016/j.drugalcdep.2005.02.002 15967595 10.1016/j.drugalcdep.2005.02.002

[77] Colzato LS, van den Wildenberg WP, Hommel B. Impaired inhibitory control in recreational cocaine users. PLoS One. 2007;2(11):e1143 doi: 10.1371/journal.pone.0001143 17989775 10.1371/journal.pone.0001143 PMC2065840

[78] Yin J, Yuan K, Feng D, Cheng J, Li Y, Cai C, et al Inhibition control impairments in adolescent smokers: electrophysiological evidence from a Go/NoGo study. Brain imaging and behavior. 2016, 10(2):497–505. doi: 10.1007/s11682-015-9418-0 26093534 10.1007/s11682-015-9418-0

[79] Nigg JT, Silk KR, Stavro G, Miller T. Disinhibition and borderline personality disorder. Dev Psychopathol. 2005;17(4):1129–49. 16613434 10.1017/s0954579405050534

[80] Ruchsow M, Groen G, Kiefer M, Buchheim A, Walter H, Martius P, et al Response inhibition in borderline personality disorder: event-related potentials in a Go/Nogo task. Journal of neural transmission. 2008;115(1):127–33. doi: 10.1007/s00702-007-0819-0 17885723 10.1007/s00702-007-0819-0

[81] Badcock JC, Michie PT, Johnson L, Combrinck J. Acts of control in schizophrenia: dissociating the components of inhibition. Psychol Med. 2002;32(2):287–97. 11866323 10.1017/s0033291701005128

[82] Enticott PG, Ogloff JR, Bradshaw JL. Response inhibition and impulsivity in schizophrenia. Psychiatry research. 2008;157(1):251–4. 17916385 10.1016/j.psychres.2007.04.007

[83] Lipszyc J, Schachar R. Inhibitory control and psychopathology: a meta-analysis of studies using the stop signal task. J Int Neuropsychol Soc. 2010;16(6):1064–76. doi: 10.1017/S1355617710000895 20719043 10.1017/S1355617710000895

[84] Nigg JT. Is ADHD a disinhibitory disorder? Psychol Bull. 2001;127(5):571–98. 11548968 10.1037/0033-2909.127.5.571

[85] Passarotti AM, Sweeney JA, Pavuluri MN. Neural correlates of response inhibition in pediatric bipolar disorder and attention deficit hyperactivity disorder. Psychiatry Res. 2010;181(1):36–43. doi: 10.1016/j.pscychresns.2009.07.002 19926457 10.1016/j.pscychresns.2009.07.002 PMC2795009

[86] Willcutt EG, Doyle AE, Nigg JT, Faraone SV, Pennington BF. Validity of the executive function theory of attention-deficit/hyperactivity disorder: a meta-analytic review. Biol Psychiatry. 2005;57(11):1336–46. doi: 10.1016/j.biopsych.2005.02.006 15950006 10.1016/j.biopsych.2005.02.006

[87] Swick D, Honzel N, Larsen J, Ashley V, Justus T. Impaired response inhibition in veterans with post-traumatic stress disorder and mild traumatic brain injury. J Int Neuropsychol Soc. 2012;18(5):917–26. doi: 10.1017/S1355617712000458 22595028 10.1017/S1355617712000458

[88] Aupperle RL, Melrose AJ, Stein MB, Paulus MP. Executive function and PTSD: disengaging from trauma. Neuropharmacology. 2012;62(2):686–94. doi: 10.1016/j.neuropharm.2011.02.008 21349277 10.1016/j.neuropharm.2011.02.008 PMC4719148

[89] Houben K, Nederkoorn C, Wiers RW, Jansen A. Resisting temptation: decreasing alcohol-related affect and drinking behavior by training response inhibition. Drug Alcohol Depend. 2011;116(1–3):132–6. doi: 10.1016/j.drugalcdep.2010.12.011 21288663 10.1016/j.drugalcdep.2010.12.011

[90] van Koningsbruggen GM, Veling H, Stroebe W, Aarts H. Comparing two psychological interventions in reducing impulsive processes of eating behaviour: effects on self-selected portion size. Br J Health Psychol. 2014;19(4):767–82. doi: 10.1111/bjhp.12075 24147757 10.1111/bjhp.12075

[91] Veling H, Aarts H, Stroebe W. Using stop signals to reduce impulsive choices for palatable unhealthy foods. Br J Health Psychol. 2013;18(2):354–68. doi: 10.1111/j.2044-8287.2012.02092.x 23017096 10.1111/j.2044-8287.2012.02092.x

[92] Houben K, Jansen A. Chocolate equals stop. Chocolate-specific inhibition training reduces chocolate intake and go associations with chocolate. Appetite. 2015;87:318–23. doi: 10.1016/j.appet.2015.01.005 25596041 10.1016/j.appet.2015.01.005

[93] Houben K, Jansen A. Training inhibitory control. A recipe for resisting sweet temptations. Appetite. 2011;56(2):345–9. doi: 10.1016/j.appet.2010.12.017 21185896 10.1016/j.appet.2010.12.017

[94] Veling H, Aarts H, Papies EK. Using stop signals to inhibit chronic dieters’ responses toward palatable foods. Behaviour research and therapy. 2011;49(11):771–80. doi: 10.1016/j.brat.2011.08.005 21906724 10.1016/j.brat.2011.08.005

[95] Lawrence NS, O'Sullivan J, Parslow D, Javaid M, Adams RC, Chambers CD, et al Training response inhibition to food is associated with weight loss and reduced energy intake. Appetite. 2015;95:17–28. doi: 10.1016/j.appet.2015.06.009 26122756 10.1016/j.appet.2015.06.009 PMC4596151

[96] Ferrey AE, Frischen A, Fenske MJ. Hot or not: response inhibition reduces the hedonic value and motivational incentive of sexual stimuli. Front Psychol. 2012;3:575 doi: 10.3389/fpsyg.2012.00575 23272002 10.3389/fpsyg.2012.00575 PMC3530044

[97] Stice E, Lawrence NS, Kemps E, Veling H. Training motor responses to food: A novel treatment for obesity targeting implicit processes. Clinical psychology review. 2016;49:16–27. doi: 10.1016/j.cpr.2016.06.005 27498406 10.1016/j.cpr.2016.06.005

[98] Allom V, Mullan B, Hagger M. Does inhibitory control training improve health behaviour? A meta-analysis. Health Psychol Rev. 2016;10(2):168–86. doi: 10.1080/17437199.2015.1051078 26058688 10.1080/17437199.2015.1051078

[99] Jones A, Di Lemma LC, Robinson E, Christiansen P, Nolan S, Tudur-Smith C, et al Inhibitory control training for appetitive behaviour change: A meta-analytic investigation of mechanisms of action and moderators of effectiveness. Appetite. 2016;97:16–28. doi: 10.1016/j.appet.2015.11.013 26592707 10.1016/j.appet.2015.11.013

[100] Turton R, Bruidegom K, Cardi V, Hirsch CR, Treasure J. Novel methods to help develop healthier eating habits for eating and weight disorders: a systematic review and meta-analysis. Neuroscience & Biobehavioral Reviews. 2016;61:132–55. 26695383 10.1016/j.neubiorev.2015.12.008

[101] Adams RC, Lawrence NS, Verbruggen F, Chambers CD. Training response inhibition to reduce food consumption: Mechanisms, stimulus specificity and appropriate training protocols. Appetite. 2017;109:11–23. doi: 10.1016/j.appet.2016.11.014 27838443 10.1016/j.appet.2016.11.014 PMC5240656

[102] Thorell LB, Lindqvist S, Bergman Nutley S, Bohlin G, Klingberg T. Training and transfer effects of executive functions in preschool children. Dev Sci. 2009;12(1):106–13. doi: 10.1111/j.1467-7687.2008.00745.x 19120418 10.1111/j.1467-7687.2008.00745.x

[103] Spierer L, Chavan CF, Manuel AL. Training-induced behavioral and brain plasticity in inhibitory control. Front Hum Neurosci. 2013;7:427 doi: 10.3389/fnhum.2013.00427 23914169 10.3389/fnhum.2013.00427 PMC3729983

[104] Wright L, Lipszyc J, Dupuis A, Thayapararajah SW, Schachar R. Response inhibition and psychopathology: a meta-analysis of go/no-go task performance. J Abnorm Psychol. 2014;123(2):429–39. doi: 10.1037/a0036295 24731074 10.1037/a0036295

[105] Bradley MM, Cuthbert BN, Lang PJ. Picture media and emotion: Effects of a sustained affective context. Psychophysiology. 1996; 33(6):662–70. 8961788 10.1111/j.1469-8986.1996.tb02362.x

[106] Pace-Schott EF, Shepherd E, Spencer RM, Marcello M, Tucker M, Propper RE, et al Napping promotes inter-session habituation to emotional stimuli. Neurobiology of learning and memory. 2011;95(1):24–36. doi: 10.1016/j.nlm.2010.10.006 20969968 10.1016/j.nlm.2010.10.006 PMC3032361

