# Supplementary material for: Do all inhibitions act alike? A study of go/no-go and stop-signal paradigms
Source: PLoS One. 2017 Oct 24;12(10):e0186774. doi: 10.1371/journal.pone.0186774 (PMC5655479; doi:10.1371/journal.pone.0186774)
Supplement: S1 Table — (DOCX) [file pone.0186774.s001.docx]

**Supporting information**

On GAPED image bank, stimuli are rated on a 0-100 valence scale, with 0 indicating the most negative valence value, 100 indicates the most positive valence value and 50 is a neutral valence value. Stimuli are also rated on a 0-100 arousal scale with 0 indicating the lowest level of arousal and 100 indicating the highest level of arousal. The valence rates of pictures selected out of GAPED were between 0 and16 for negative stimuli, and between 40 and 60 for neutral stimuli. The two groups of pictures differed significantly from one another both in valence [mean negative = 8.63, mean neutral = 53.74; *t*(28) = -43.23, *p* < .001] and arousal [mean negative = 75.91, mean neutral = 21.78; *t*(28) = 31.26, *p* < .001] rates.

**S1 Table. Valence and Arousal Means for Images Selected out of GAPED Image Bank**

|  | Negative | Neutral | *P* |
| --- | --- | --- | --- |
| Valence | 8.63 | 53.74 | < .001 |
| Arousal | 75.91 | 21.78 | < .001 |
