# Supplementary material for: Do all inhibitions act alike? A study of go/no-go and stop-signal paradigms
Source: PLoS One. 2017 Oct 24;12(10):e0186774. doi: 10.1371/journal.pone.0186774 (PMC5655479; doi:10.1371/journal.pone.0186774)
Supplement: S2 Table — (DOCX) [file pone.0186774.s002.docx]

**Supporting information**

On IAPS image bank, stimuli are rated on a 1-9 scale, with 1 indicating the most negative valence value, 9 indicating the most positive valence value and 5 is a neutral valence value. Stimuli are also rated on a 1-9 arousal scale with 1 indicating the lowest level of arousal and 9 indicating the highest level of arousal. The valence rates of pictures selected from IAPS were between 1 and 2.5 for negative stimuli and between 4 and 6 for neutral stimuli. Two groups of pictures differed significantly from one another both in valence [mean negative = 1.94, mean neutral = 5.11; *t*(75) = -47.96, *p* < .001] and arousal [mean negative = 6.21, mean neutral = 3.3; *t*(75) = 29.97, *p* < .001] rates.

**S2 Table. Valence and Arousal Means for Images Selected out of IAPS Image Bank**

|  | Negative | Neutral | *P* |
| --- | --- | --- | --- |
| Valence | 1.94 | 5.11 | < .001 |
| Arousal | 6.21 | 3.3 | < .001 |
