# Supplementary material for: Do all inhibitions act alike? A study of go/no-go and stop-signal paradigms
Source: PLoS One. 2017 Oct 24;12(10):e0186774. doi: 10.1371/journal.pone.0186774 (PMC5655479; doi:10.1371/journal.pone.0186774)
Supplement: S3 Table — (DOCX) [file pone.0186774.s003.docx]

**Supporting information**

**S3 Table. An overview of the selected images.**

An overview of the pictures selected out of IAPS

*Neutral:* 2005, 2038, 2102, 2191, 2200, 2206, 2210, 2214, 2273, 2359, 2372, 2382, 2383, 2384, 2385, 2390, 2393, 2411, 2435,2446, 2480, 2488, 2489, 2506, 2512,2514, 2521, 2525, 2535, 2570, 2575, 2593, 2620, 5390, 5395, 5720, 7000, 7001, 7002, 7003, 7004, 7006, 7009, 7010, 7011, 7012, 7013, 7014, 7016, 7017, 7018, 7019, 7020, 7021, 7025, 7026, 7030, 7031, 7032, 7033, 7034, 7035, 7036, 7037, 7038, 7039, 7040,7041, 7042, 7043, 7044, 7045, 7046, 7050, 7052, 7053, 7054, 7055, 7056, 7057, 7059, 7060, 7061, 7062, 7077, 7080, 7081, 7090, 7092, 7095, 7096, 7100, 7110, 7130, 7137, 7140, 7150, 7160, 7161, 7170, 7175, 7179, 7180, 7182, 7183, 7184, 7186, 7187, 7188, 7190, 7192, 7205, 7207, 7211, 7217, 7224, 7233, 7234, 7235, 7236, 7237, 7300, 7490, 7491, 7500, 7546, 7560, 7595, 7705

(Mean valence = 5.09; mean arousal = 3.3)

*Negative:* 2053, 2345.1, 2703, 2799, 2800, 2811, 3000, 3005.1, 3010, 3015, 3016, 3017, 3030, 3051, 3053, 3060, 3061, 3062, 3063, 3064, 3068, 3069, 3071, 3080, 3100, 3102, 3110, 3120, 3130, 3150, 3168, 3170, 3180, 3181, 3220, 3225, 3261, 3266, 3301, 3350, 3400, 3550.1, 6021, 6022, 6313, 6315, 6350, 6415, 6510, 6520, 6560, 6563, 6838, 9075, 9140, 9181, 9252, 9253, 9300, 9301, 9325, 9326, 9332, 9405, 9410, 9412, 9413, 9414, 9420, 9433, 9570, 9571, 9635.1, 9800, 9901, 9921

(Mean valence = 1.94; mean arousal = 6.21)

An overview of the pictures selected out of GAPED

*Neutral:* N006, N010, N011, N014, N018, N022, N024, N031, N033, N034, N035, N037, N046, N061, N062, N065, N067, N068, N072, N073, N079, N085, N087, N089, N091, N095, N096, N098, N111

(Mean valence = 53.74; mean arousal = 21.78)

*Negative:* A007, A025, A030, A033, A036, A041, A068, A075, A083, A089, A091, A095, A100, A104, A117, A125, H005, H034, H037, H041, H064, H077, H087, H122, Sn048, Sn124, Sp051, Sp081, Sp153

(Mean valence = 8.63; mean arousal = 75.91)
